# Eg5 steps it up!

**DOI:** 10.1186/1747-1028-1-31

**Published:** 2006-12-15

**Authors:** Megan T Valentine, Polly M Fordyce, Steven M Block

**Affiliations:** 1 Department of Biological Sciences, Stanford University, Stanford CA 94305, USA; 2 Department of Physics, Stanford University, Stanford CA 94305, USA; 3 Department of Applied Physics, Stanford University, Stanford CA 94305, USA

## Abstract

Understanding how molecular motors generate force and move microtubules in mitosis is essential to understanding the physical mechanism of cell division. Recent measurements have shown that one mitotic kinesin superfamily member, Eg5, is mechanically processive and capable of crosslinking and sliding microtubules *in vitro*. In this review, we highlight recent work that explores how Eg5 functions under load, with an emphasis on the nanomechanical properties of single enzymes.

## Review

### Eg5 motors slide microtubules during cell division

**Figure 1:**
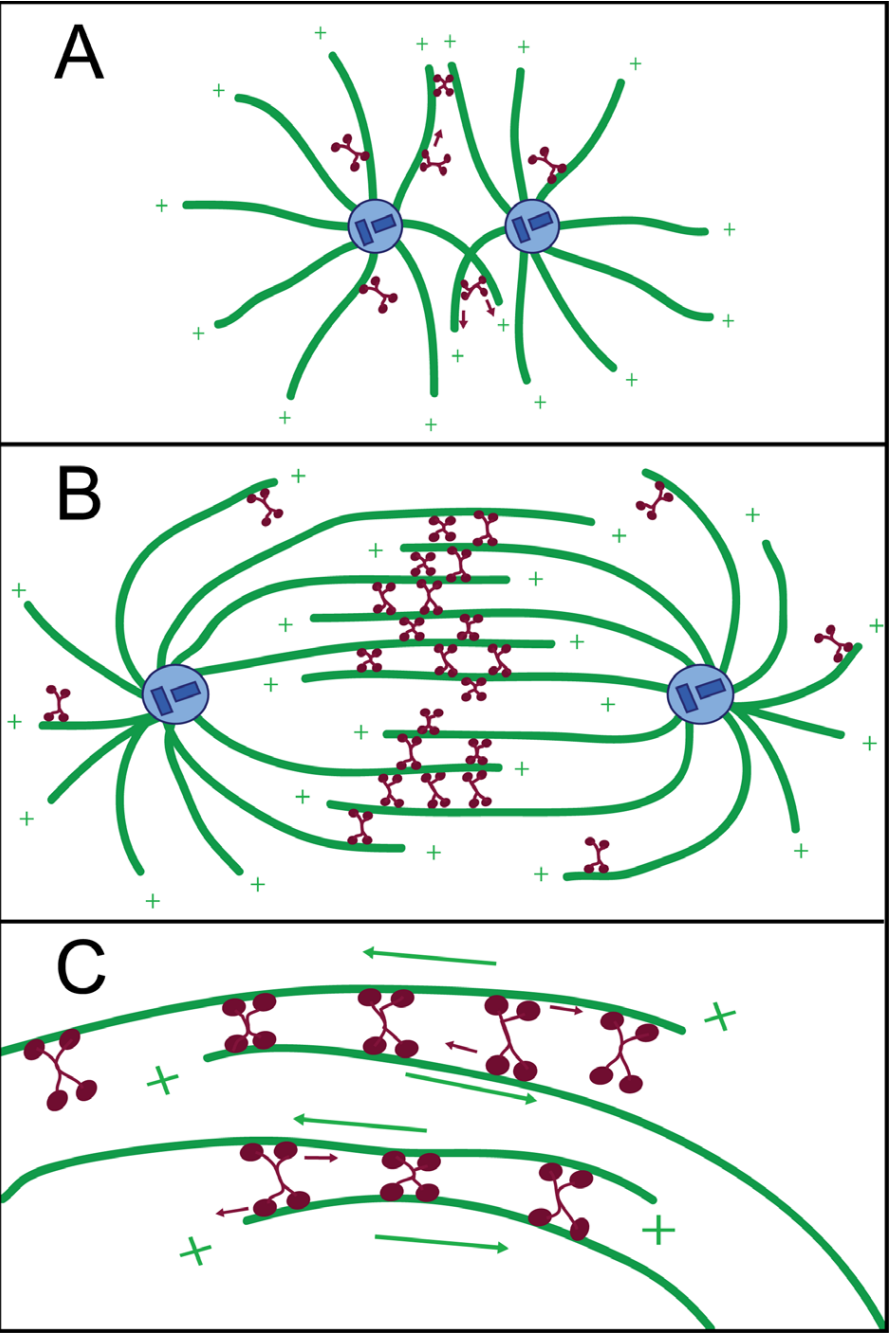
**Schematic depicting Eg5 activity in the mitotic spindle**. Tetrameric Eg5 motors (red) help organize microtubules (green) to form the mitotic spindle. (A) At the onset of mitosis, the duplicated centrosomes (blue) separate and nucleate two microtubule asters. Processive Eg5 motors may translocate to the plus-ends of microtubules, located distal to the centrosomal organizing center and by crosslinking antiparallel microtubules, may promote bipolarity. (B) By metaphase, a stable bipolar spindle has formed. Eg5 motors likely provide structural integrity and also slide microtubules toward the centrosomes, contributing to the generation of poleward flux. (C) A close-up depiction of Eg5 motors walking to the plus ends of antiparallel microtubules, moving both poleward simultaneously.

Eg5, a member of the Kinesin-5 subclass of kinesins, is a plus-end-directed tetrameric kinesin-family protein that influences the assembly and organization of the mitotic spindle, a self-assembled and dynamic microtubule-based structure that orchestrates chromosome segregation in dividing cells (Figure 1) [1-3]. Eg5 action is essential: when it is depleted from the cytoplasm of meiotically-mature *Xenopus laevis *eggs, abnormal monopolar spindles form, preventing successful division. Homologous proteins (referred to generically as 'Eg5' herein) with similar loss-of-function phenotypes have been identified across organisms [4-7].

During metaphase, the mitotic spindle maintains constant size and shape despite poleward movement of microtubules that is coupled to minus-end disassembly at the spindle pole, a process known as "poleward flux" [8,9]. The mechanism driving poleward translocation remains controversial, but likely involves both microtubule polymerization in the mid-zone as well as motor-mediated microtubule sliding [10-12].

The tetrameric structure of Eg5 makes it a particularly attractive candidate for binding antiparallel microtubules and sliding them apart [13,14]. However, dissecting the role of Eg5 in poleward flux is challenging, since its selective removal or inhibition often leads to serious mitotic defects. In *Xenopus *egg extracts, experiments are tractable as it is possible to stabilize bipolar spindles while monitoring the movement of microtubules using fluorescence speckle microscopy [11,15]. Such experiments have indicated that Eg5 is required for poleward translocation of spindle microtubules [16]. Moreover, biochemical depletion of Eg5 significantly decreases flux rate, and pharmacological inhibition of Eg5 produces a dose-dependent slowing [11]. Flux persists in spindles in which microtubule depolymerization has been blocked through chemical treatment by hexylene glycol or the addition of pole-disrupting reagents, providing further evidence that Eg5-mediated sliding, and not depolymerization, dominates flux generation in egg extracts [16,17].

While Eg5 may be essential for generating flux in *Xenopu*s-derived spindles, where flux is fast relative to chromosome movement, its role in higher eukaryotes, where flux is relatively slow, is less clear [18,19]. Inhibition of Eg5 in mammalian PtK1 cells results in only a minor reduction in the flux rate, suggesting that depolymerization may be more important in this system [20]. Consistent with this, depletion of the Kinesin-13 subfamily depolymerizing proteins KIF2A and MCAK in human U2OS cells eliminated poleward flux and reduced poleward chromosome velocity at anaphase, albeit with no deleterious effect on overall mitotic progression [19]. Flux rates vary significantly among different cell types and between mitotic and meiotic systems, suggesting there may not be a single dominant mechanism or function for poleward flux in all cells [8,18,21].

### Establishing Eg5 processivity is critical to understanding its function

Intriguingly, the distribution of Eg5 in *Xenopus *egg extracts is static with respect to poleward-fluxing microtubules. Two competing models have been proposed to explain this effect. In the first, ensembles of Eg5 motors transiently bind to a microtubule, stroke, and detach, thereby pushing microtubules poleward without maintaining prolonged contact with the tubulin substrate. To prevent diffusion away from the spindle, Eg5 motors are proposed to interact with a non-microtubule-based matrix in the spindle [22-24]. Although the molecular identity of the proposed "spindle matrix" is unknown, several candidate filaments are required for spindle function, including the nuclear/mitotic apparatus protein (NuMA) [25], lamin-B [26], and the branched polyelectrolyte, poly(ADP-ribose) [27]. Alternatively, if Eg5 tetramers are mechanically processive, taking multiple steps along the microtubule before detaching, they could simultaneously move towards the plus ends of the two antiparallel microtubules they crosslink [22]. This would slide both microtubules toward opposing poles while the Eg5 motors remained stationary, as if walking on juxtaposed treadmills.

The key distinction between these models is the amount of time Eg5 motors remain bound to the microtubule during the kinetic cycle. Early solution biochemistry experiments sought to resolve this controversy by measuring the "chemical processivity", or the number of ATP molecules consumed per diffusional encounter with the microtubule, which scales with the ratio of the rate of catalysis, *k*_cat_, to the equilibrium binding constant, *K*_50%MTs _[28]. Dimeric *Xenopus*-derived truncation mutants were found to be less chemically processive than either kinesin monomers or ncd dimers, both of which are known to be mechanically nonprocessive. From these data, it was concluded that Eg5 is "slightly if at all processive." The dose-responsive hyperbolic slowing of flux in response to pharmacological inhibition of Eg5 [11] – resembling the slowing of gliding actin filaments when the number of driving nonprocessive myosin motors is reduced- was taken as further evidence of nonprocessivity [29]. Although these data have been widely interpreted as evidence of nonprocessivity, a lack of mechanical data precluded a definitive determination.

**Figure 2:**
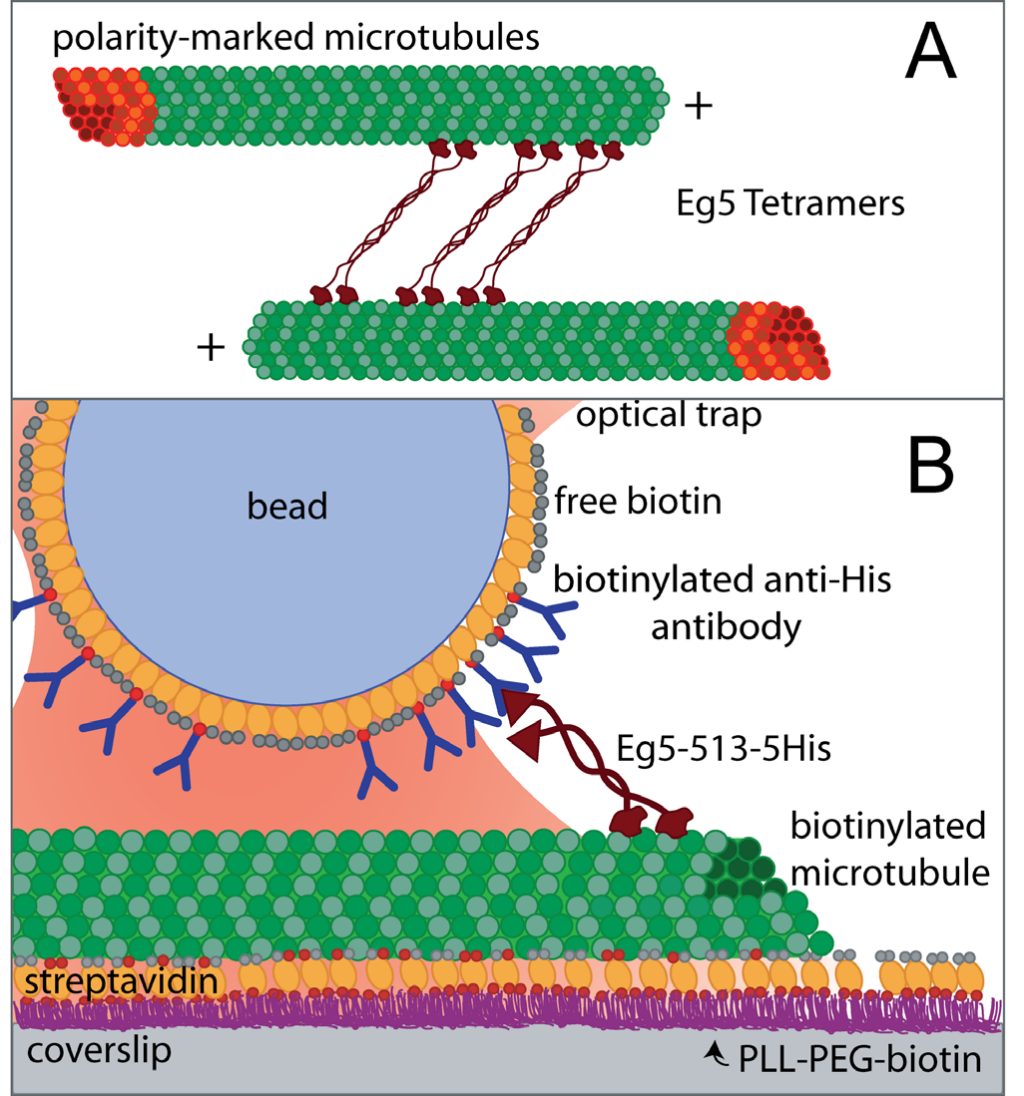
**Schematic showing *in vitro *assay designs for Eg5 motor studies**. (A) Depiction of a fluorescence-based assay used to demonstrate purified full length Eg5 tetramers are capable of crosslinking and sliding microtubules *in vitro *[13]. Unlabeled Eg5 motors bind to fluorescent, polarity-marked microtubules, causing the microtubules to slide apart. (B) Schematic showing optical trapping assay used to observe processive movement of Eg5 dimers [30]. His-tagged motors are attached to streptavidin-coated beads through a biotinlyated PentaHis antibody. Coverslip surfaces are precoated with poly-L-lysine-*graft-*poly(ethylene glycol) polymers to prevent surface-induced denaturation of Eg5 at the glass interface. Polymers are biotinylated to allow the specific attachment of biotinlyated microtubules via a streptavidin linkage.

A clever *in vitro *fluorescence assay demonstrated that full length Eg5 tetramers, in the absence of secondary matrix proteins, were capable of simultaneously binding two microtubules and moving toward the plus-ends of both, once again raising the possibility of mechanical processivity and reviving the debate (Figure 2A) [13]. This study provided the first direct evidence that purified Eg5 motors were capable of providing structural integrity and motive force to microtubules. For efficient sliding, it seemed possible that Eg5 motors remained microtubule-bound for sustained periods; however, these experiments were performed under multiple motor conditions, so the mechanical processivity of single motors remained unresolved.

**Figure 3:**
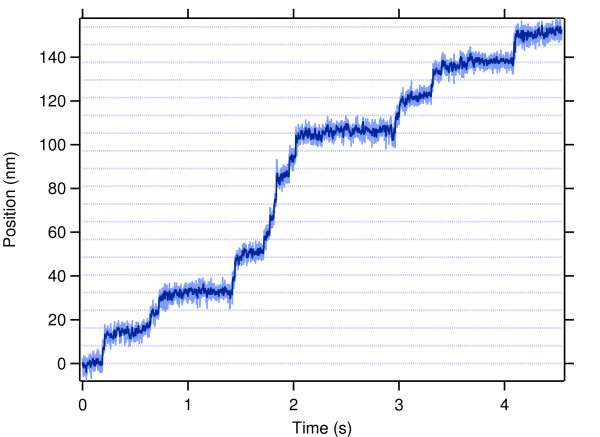
**Representative trace of position of single Eg5 dimer moving *in vitro***. Record shows motion of a bead-attached Eg5 dimer held in an optical trap, and walking along microtubules in 8.1-nm steps. Position (light blue) and smoothed position (dark blue) are plotted as a function of time; dotted lines are placed every 8.1 nm to guide the eye. Experimental conditions: 2 mM ATP, 4 pN load applied toward the microtubule plus-end (assisting motion).

Optical trapping measurements allow the direct observation of individual motors as they move along a microtubule and provide a definitive measurement of mechanical processivity. Although extensively used to characterize the biophysical properties of conventional kinesin, optical trapping assays have not been applied widely to other kinesin-related proteins, largely because traditional assays rely on especially fortuitous surface interactions specific to conventional kinesin. Measurements of mitotic kinesins require the development of new assays using polymer-coated surfaces and stereospecific attachment schemes to create robust, functionalized and protein-resistant surfaces [30-32]. Using one such *in vitro *assay (Figure 2B), it was shown that individual dimeric human Eg5 proteins walk processively, taking 8 steps on average before dissociation [30]. Frequent runs of multiple steps were observed with clear transitions between each step (Figure 3), and statistical tests verified that single motors were sufficient to power movement. As expected for a processive enzyme, the step size is 8.1 nm, identical to that of conventional kinesin and the spacing between tubulin heterodimers in the microtubule lattice. A subsequent study using single-molecule fluorescence confirmed that full-length GFP-tagged Eg5 tetramers move processively on microtubules as well [33].

In light of these single-molecule experiments, previous conclusions of Eg5 nonprocessivity must be reconsidered. Chemical processivity measurements should never be mistaken as evidence of true mechanically-processive movement [34], and may be particularly poor estimators for motors with small run lengths. The hyperbolic slowing of Eg5-driven flux as a function of inhibitor concentration that was initially interpreted as evidence of nonprocessivity may instead indicate that inhibited motors remain weakly associated with microtubules, acting as a brake [33,35,36]. Further experiments investigating the mechanical basis of inhibition will be required to fully understand this result. While Eg5's mechanical processivity and ability to crosslink and slide microtubules *in vitro *certainly does not rule out the presence of a static spindle matrix, the immobilization of Eg5 within the spindle can no longer be used as evidence supporting its existence.

### Eg5's load-dependent mechanochemistry shows key distinctions as compared to conventional kinesin

Establishing that Eg5 is a processive enzyme not only sheds new light on its physiological role, but readily allows measurement of some of the most interesting and informative motor properties: the force-velocity relationship and stall force. Taken together, the mechanical properties of Eg5 show several important distinctions from those of conventional kinesin, a cargo transporter. Through a comparative analysis we may begin to gain insight into how physiology influences motor function, and how small changes in protein structure or organization give rise to the distinct mechanical properties of all kinesin family members.

Both Eg5 and kinesin velocities display a Michaelis-Menten dependence on ATP concentration, but the overall velocity of Eg5 is significantly slower, with a maximal stepping rate of 100 nm/s, compared to approximately 650 nm/s for conventional kinesin [30,37]. Eg5 is also much less processive, taking ~8 steps at a time at saturating ATP and zero load, while kinesin takes 50 steps or more under similar conditions [30,38].

**Figure 4:**
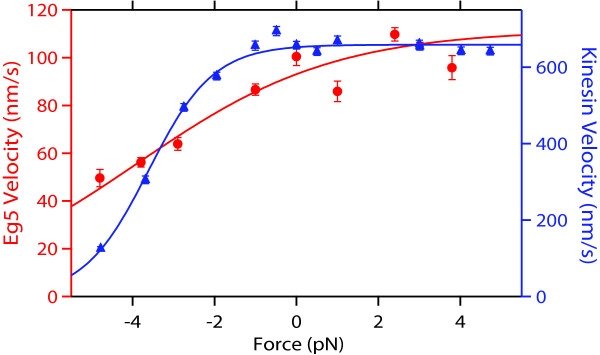
**Comparison of the force-dependence of the velocities of Eg5 and conventional kinesin**. Eg5 (red, left axis) [30] and conventional kinesin (blue, right axis) [37] velocity as a function of force, as measured with a force-clamped optical trap. Positive forces indicate that load was applied toward the plus-end of the microtubule, assisting motion; negative forces hinder translocation. Conventional kinesin slows much more dramatically than Eg5 does under hindering load.

The most significant difference between the two motors lies in their response to applied force. At fixed ATP conditions, both kinesin and Eg5 velocities remain roughly constant for assisting loads and slow monotonically for hindering loads; however, while kinesin slows by a factor of ~8 from its maximal value at -5 pN, Eg5 is significantly less sensitive to force, slowing by only a factor of three (Figure 4) [30,37]. While both kinesin and Eg5 dimers can sustain hindering loads as high as -7 pN, kinesin motors tend to stall and step backwards, maintaining their grip, whereas Eg5 dimers dissociate, making collection of data above -5 pN difficult [30,37,39]. Eg5 motors may eventually stall at extremely high loads: a linear extrapolation of the force-velocity curve to zero velocity suggests the stall force would be approximately -9 pN, near the theoretical maximum allowed for work produced by the hydrolysis of ATP [40]. Future studies at higher forces, inspired by recent work that probed the effect of the sudden application of large superstall loads to conventional kinesin [39], may be required to probe this regime and may reveal additional information about Eg5 mechanochemistry.

### Structural differences may be responsible for unusual force response

Several recent studies have revealed structural properties that could contribute to Eg5's unique mechanochemical characteristics. The crystal structure of the motor domain for a human-derived Eg5 monomer bound to ADP showed a novel, ordered neck linker configuration that is docked perpendicular to the long edge of the protein via a series of hydrogen bonds [41]. In all previous kinesin family member structures, the neck linker was either disordered or docked parallel to the long edge of the protein, and in the ADP-bound state, the neck linker is typically floppy [42-45]. The residues involved in Eg5's novel docking are highly conserved among Kinesin-5 family members, suggesting that this conformation may be specific to this subclass of motors [41].

Although a rigid neck linker would impart molecular stiffness, perhaps allowing controlled microtubule sliding under constant tension, it could also hinder each motor head's diffusional search for the next tubulin binding site. A unique mode of motility might provide a much-needed mechanical compromise, allowing processive motion under significant load even with an inflexible neck linker. Based on a series of ensemble FRET measurements, Rosenfeld, *et al*. proposed three different rigid neck linker conformations for the ATP-, ADP- and no nucleotide-bound states, and further showed that Eg5 likely moves in two sequential steps [46]. First, ATP binding docks the neck linker parallel to the motor domain, then, upon hydrolysis, the entire motor domain rolls forward along the microtubule. This two-step mode of motility could be critical for Eg5 function and may influence its relative insensitivity to applied force.

Interestingly, a recent report indicates that full-length GFP-tagged Eg5 motors display an unusual mode of motility: processive directional movement interrupted by periods of diffusion as tetramers move along a single coverslip-bound microtubule [33]. One-dimensional diffusion along the tubulin lattice has been reported for other kinesin-related proteins such as monomeric KIF1A [47,48] and MCAK, a centromere-associated depolymerizer [49]; localization arises from the electrostatic attraction of the motor to the highly negatively-charged microtubule. Further experiments will be required to determine the role of electrostatic interactions in Eg5, to demonstrate whether the diffusive state is specific to tetrameric motors moving along an isolated microtubule or exists for dimers or tetramers bridging two microtubules, and to determine how much force motors undergoing diffusion can withstand before they detach. This diffusive mode could be important to Eg5's function by increasing the total time each motor remains localized to the spindle and therefore the likelihood that tetramers will form crosslinks in cells.

### Coordination within and among Eg5 motors

Some of the biggest questions about Eg5 motility surround coordination: Do individual Eg5 dimers move by a hand-over-hand mechanism, like conventional kinesin does [50-52]? Are the four motor heads of single Eg5 tetramers coordinated? Finally, do ensembles of Eg5 motors work together to generate force in cell division, and how is the force generated by Eg5 motors balanced against forces generated by other spindle motors (such as ncd, dynein, and the chromokinesins) [53-55]?

Conventional kinesin likely works in isolation to tote cargo long distances in cells. Such highly processive movement requires that the catalytic cycles of the two motor heads be tightly coordinated to prevent simultaneous dissociation. It is proposed that intramolecular strain transmitted by the neck linker regulates the biochemical state of each motor head, keeping the two heads biochemically out-of-phase and allowing one head to maintain a tight grip on the microtubule at all times, even under considerable load [56-58].

By contrast, Eg5 probably works in small ensembles. The limited processivity of Eg5 may arise because ensembles of motors must work together within the spindle. By taking multiple steps, single motors maintain sustained contact with their microtubule substrates and aid in *de novo *spindle assembly. By dissociating quickly, however, motors detach from the microtubule before stalling and slowing other motors in the ensemble, promoting efficient sliding once spindles are formed. These short run lengths may prove to be a consequence of reduced head-head coordination resulting from the rigid nature of the neck linker. Additional experiments will be required to unravel the extent of catalytic coordination, and determine if Eg5 dimers walk hand-over-hand.

In native tetramers, another level of coordination is possible: the opposing pairs of motors heads located at either end of the coiled-coil stalk could cooperate to enhance processivity. Based on the average run length for the dimer, and assuming each pair of dimers in the homotetramer moves independently, the tetramer should remain attached to the spindle for ~64 steps, on average [30]. This simple model would predict a run length of ~520 nm, surprisingly similar to the ~580-nm average run length of GFP-Eg5 tetramers moving along a single microtubule *in vitro *[33]. In principle, either linear or torsional strain within the extended stalk domain could allow the pairs of dimers to communicate, thereby modulating tetrameric run lengths. Direct mechanical measurements of single full-length Eg5 tetramers moving on two microtubules will be necessary to probe this possibility. Finally, new *in vitro *assays capable of measuring the forces exerted by ensembles of motors are required to fully understand how mixed populations of motors work together to organize and move microtubules in cells.

## Outlook

Single-molecule measurements of the motor proteins that generate force during mitosis are indispensable for elucidating the physical basis of cell division. Establishing whether or not these motors are processive and how they respond to force is critical to developing predictive computational models and to understanding how ensembles of motors cooperate to balance forces during each stage of division. Although many mitotic motor proteins have been identified, little nanomechanical characterization has been performed and many important questions remain. The new *in vitro *assays [13,30] reviewed here should allow complete characterization of Kinesin-5 subclass members, as well as rapid expansion into new classes of motor proteins. These new data will permit unprecedented comparative studies of diverse kinesin family members and shed new light on both how protein structure influences motor function and how biochemical energy is harnessed into productive work by all mechanoenzymes.

## Competing interests

The author(s) declare that they have no competing interests.
